# Minimum Electromyographic Burst Duration in Healthy Controls: Implications for Electrodiagnosis in Movement Disorders

**DOI:** 10.1002/mdc3.13044

**Published:** 2020-08-26

**Authors:** Alexis F. Collins, Steven T.R. Brown, Mark R. Baker

**Affiliations:** ^1^ Translational and Clinical Research Institute, The Medical School Newcastle University Newcastle upon Tyne United Kingdom; ^2^ Sheffield Institute for Translational Neuroscience The University of Sheffield Sheffield United Kingdom; ^3^ Department of Neurology Royal Victoria Infirmary Newcastle upon Tyne United Kingdom; ^4^ Department of Clinical Neurophysiology Royal Victoria Infirmary Newcastle upon Tyne United Kingdom

**Keywords:** EMG, functional movement disorders, myoclonus

## Abstract

**Background:**

Electromyogram (EMG) burst duration can provide additional diagnostic information when investigating hyperkinetic movement disorders, particularly when a functional movement disorder is suspected. It is generally accepted that EMG bursts <50 milliseconds are pathological.

**Objective:**

To reassess minimum physiological EMG burst duration.

**Methods:**

Surface EMG was recorded from face, trunk, and limb muscles in controls (n = 60; ages 19–85). Participants were instructed to generate the briefest possible ballistic movements involving each muscle (40 repetitions) or, in muscles spanning joints, to generate rapid rhythmic alternating movements (20–30 seconds), or both.

**Results:**

We found no effect of age on EMG burst duration. However, EMG burst duration varied significantly between body regions. Rhythmic EMG bursts were shorter than ballistic bursts but only significantly so for lower limbs (*P* < 0.001). EMG bursts of duration <50 milliseconds were frequently observed, particularly in appendicular muscles.

**Conclusion:**

We present normal reference data for minimum EMG burst duration, which may assist clinical interpretation when investigating hyperkinetic movement disorders.

Electrophysiological investigations are widely used in the diagnosis of hyperkinetic movement disorders, most notably tremor and myoclonus,^1^, ^2^ where they may be helpful in identifying patients with functional hyperkinetic movement disorders. Electromyography, which is a commonly available modality, is perhaps the least challenging of the electrophysiological techniques in terms of data acquisition. Although there is increasing interest in the application of complex signal processing techniques to such data (eg, refs. 3, 4), analysis limited to the simple parameter of electromyogram (EMG) burst duration is widely used to guide diagnosis (and localization) of myoclonus^5^ and has the additional advantage of being simple to measure. Typically, EMG is recorded simultaneously with video‐electroencephalography and only a limited number of channels are available for EMG (sometimes a single channel). The placement of EMG electrodes is then determined by the number available and the clinician's assessment as to which muscles are most involved. Surprisingly, despite the widespread application of EMG burst duration in clinical practice, normal values are only available from small studies and only for a limited selection of appendicular muscles.

Sampling EMG burst duration for a ballistic movement generated by an individual muscle in a large population of healthy controls to determine normal limits for burst duration based on mean and standard deviation would be one approach. However, because the individual discrete EMG bursts contributing to the triphasic agonist–antagonist–agonist EMG pattern of ballistic limb movements cannot always be distinguished visually,^6^ ballistic EMG bursts appear artificially prolonged (ie, composites of the first and second agonist bursts), thus skewing the distribution of data. To address this, where possible, it is recommended that EMG is recorded from the antagonist muscle acting at the same articulation to exclude contaminated agonist bursts from analysis. However, this is not always possible, particularly where there is no antagonist muscle (eg, facial muscles) or where clean EMG recording from an antagonist muscle is difficult to achieve noninvasively (eg, abdominal muscles). To circumvent this problem, the approach adopted has therefore been to simply measure the shortest EMG burst duration, rather than the mean, and interpret this with reference to a lower limit for the duration of normal physiological EMG bursts.

Although there is general agreement in the literature that short‐duration EMG bursts are pathological, there is debate as to the upper limit, with some accepting a burst duration of <70 milliseconds^1^, ^7^ and others advocating a cut‐off of <50 milliseconds.^5^, ^8^, ^9^ By contrast, in functional movement disorders (FMDs), which are thought to arise as a consequence of abnormal predictive coding by the brain^10^ and thus by definition remain constrained by physiological mechanisms of movement control, the duration of EMG bursts observed, particularly in functional myoclonus, should be comparable with those generated by voluntary ballistic movements; based on data collated from a number of small studies, this appears to be consistently longer than 50 milliseconds in duration (eg, neck,^11^ upper limb^6^, ^12^, ^13^, ^14^ lower limb^15^).

Here we have addressed the need for a more comprehensive data set of normal values for minimum EMG burst duration by measuring the minimum voluntary EMG burst duration in cranial, axial, and appendicular muscles in healthy controls instructed to produce brief ballistic voluntary contractions (to mimic myoclonus) or rhythmic contractions (mimicking tremor) in these muscles. As a reference resource, these data should assist clinicians in the electrodiagnostic investigation of complex hyperkinetic movement disorders.

## Methods

### Participants

Experiments were conducted in 60 healthy volunteers (age range, 19–85; mean age, 34; 30 women). Participants were excluded if they had a neurological disorder or an implanted device (eg, cardiac pacemaker). Experiments were approved by the Newcastle University Ethics Committee and conformed to the Declaration of Helsinki. All participants provided written informed consent.

Analysis also included anonymized EMG data acquired from patients with movement disorders (n = 3) referred for neurophysiologic investigation as part of a routine diagnostic workup and identified by a retrospective case note review.

### Recordings

Referential surface EMG recordings were made using adhesive (Ag/AgCl) electrodes attached to the skin overlying cranial (temporalis, orbicularis oculi, risorius), trunk (trapezius, infraspinatus, rhomboids, latissimus dorsi, pectoralis major, rectus abdominis superior, rectus abdominis inferior), upper limb (deltoid, triceps brachii, biceps brachii, extensor digitorum communis, flexor carpi ulnaris, abductor pollicis brevis, first dorsal interosseous), and lower limb (vastus lateralis, biceps femoris, tibialis anterior, medial gastrocnemius, extensor digitorum brevis, abductor hallucis) muscles. The anode (reference electrode) was placed on the bony prominence or tendon and the cathode (active electrode) on the muscle belly (approximate interelectrode distance of 3 cm). Consistent EMG electrode placement is important when comparing EMG burst durations across individuals; if the electrode spacing is too large, it becomes in essence a monopolar recording (with the reference electrode behaving as an indifferent), which could reduce the duration of the recorded EMG burst. Surface EMG signals were amplified (5 K) and filtered (3 Hz–2 KHz) using an 8‐channel Digitimer D360 amplifier (Digitimer Ltd, Welwyn Garden City, UK), controlled via a dedicated laptop (the sampling frequency should ideally be set at four times the low pass filter frequency). The output from the D360 was connected to an analog to digital converter (Cambridge Electronic Design Micro 1401; Cambridge Electronic Design Ltd, Cambridge, UK) and signals digitized at a sampling frequency of 5 KHz. EMG signals could be viewed on the data acquisition laptop using dedicated software (Spike2; Cambridge Electronic Design Ltd).

### Behavioral Tasks

Once all surface EMG electrodes were in position, participants were asked to contract the individual muscles separately. Participants were provided with guidance as to the optimum method for activating individual muscles for each task.

#### Mimicking Brief Myoclonic Jerks

For this task, participants made ballistic movements (brief muscle contractions generated with maximum velocity and acceleration) such that the EMG exhibited high motor unit firing rates. The instruction given to participants for each muscular contraction was that it should be as brief and rapid as possible (thus mimicking myoclonic movements). Where necessary, the investigator also provided a demonstration to the participants of what was required. For proximal and axial/trunk muscles, participants were shown an anonymized video of the movement required.

#### Mimicking Repetitive Myoclonic/Tremulous Movements

For articular muscles (ie, muscles spanning joints in the limbs), participants were also asked to make self‐paced (ie, without external feedback) rhythmic alternating (oscillating) movements as rapidly as possible, thus mimicking tremor.

In the interests of time, participants were separated into 3 experimental subgroups, with each subgroup testing a set of different muscles. Recordings from the upper body were made from a total of 18 volunteers; cranial, facial, arm, and foot muscles were tested in 20 participants, and lower limb muscle contractions were recorded in 36 participants. There was some overlap of participants for the 3 experiments. Each muscle was recorded for a duration of 20 to 30 seconds (~40 muscle contractions) while the volunteers executed the movements. Each contraction task was separated by 1 to 2 minutes of rest.

#### Data Analysis

**FIG 1:**
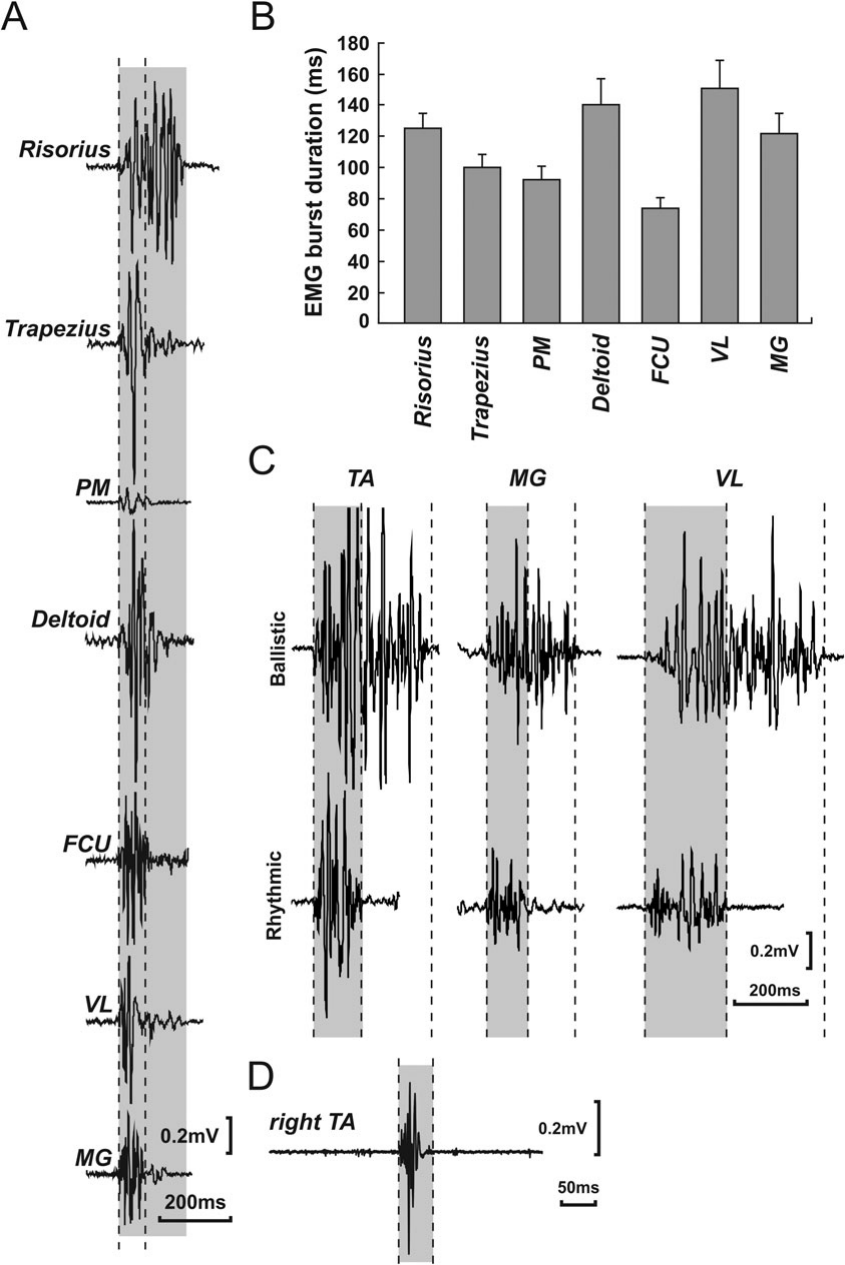
(**A**) Raw EMG data. Examples of unrectified raw surface EMG illustrating typical EMG bursts acquired from cranial (risorius), shoulder (trapezius, PM, deltoid), upper limb (FCU), and lower limb (VL, MG) muscles on the right. The gray box shows the duration of the longest EMG burst (risorius) for comparison. (**B**) Summary bar graph. Minimum EMG burst duration was averaged for each muscle across all participants (cranial, back, and upper limb muscles [n = 20]; PM [n = 18]; lower limb muscles [n = 36]). Error bars show standard error. (**C**) Ballistic and rhythmic EMG bursts. Examples of unrectified surface EMG recorded from lower limb muscles in the same participant (participant 6) while making either brief voluntary ballistic muscle contractions or rapid alternating rhythmic movements. Note that EMG burst duration in rhythmic movements was consistently reduced. Vertical dashed lines indicate onset and offset of EMG bursts and gray boxes highlight the duration of the shorter rhythmic EMG bursts. (**D**) Example of a ballistic EMG burst < 50 milliseconds recorded from the tibialis anterior muscle. The gray box and dashed vertical lines here delimit a 50‐millisecond time window. EMG, electromyogram; FCU, flexor carpi ulnaris; MG, medial gastrocnemius; PM, pectoralis major; TA, tibialis anterior; VL, vastus lateralis.

EMG data were first inspected visually and any data contaminated by noise excluded from the analysis. Unrectified EMG was then reviewed and a preliminary analysis of burst duration completed in Spike2 by assigning onset and offset markers by eye (as illustrated in Fig. 1). This approach to measurement will inevitably introduce human error into the analysis, affecting accuracy (by increasing bias), precision, and stability. Measurement of burst duration was therefore automated using custom scripts compiled in Matlab (Mathworks, Natick, MA) as follows: EMG was full‐wave rectified, EMG burst onset/offset markers were assigned when the EMG amplitude increased by 2 standard deviations above baseline for both onset and offset, burst duration was then measured between burst onset and burst offset markers, and an average burst duration was determined for each muscle in each participant. The outputs from this process were again reviewed by eye, and where bursts were detected erroneously or where co‐contraction was observed by reviewing the antagonist EMG recording simultaneously, outputs were rejected.

The Shapiro‐Wilk test was used to check for normality. The Wilcoxon signed‐rank test was used for nonparametric data (null hypothesis rejected if *P* < 0.05). Where correlations between data were explored, a Spearman rank correlation coefficient was used.

## Results

EMG recordings were obtained from 60 participants (age range, 19–85; mean age, 34; 30 women) across a total of 23 muscles. Examples of typical unrectified EMG burst recordings are shown in Figure 1A, illustrating typical EMG bursts acquired from cranial (risorius), shoulder (trapezius, PM, deltoid), upper limb (FCU), and lower limb (vastus lateralis, medial gastrocnemius) muscles on the right. The width of the gray box in Figure 1A shows the duration of the longest EMG burst (risorius) for ease of comparison with other EMG bursts.

Inspection of Figure 1A would suggest that there are regional differences in the duration of EMG bursts. For example, the forearm flexor muscle illustrated appears to generate the shortest EMG burst and the facial muscle the longest, whereas proximal upper limb and lower limb muscles generate EMG bursts of comparable but intermediate durations.

Figure 1B plots the mean EMG burst duration (and standard error) for representative regional muscles averaged across subjects (cranial, trunk, and upper limb muscles [n = 20]; PM [n = 18]; lower limb muscles [n = 36]), showing a different pattern of EMG burst durations from that illustrated in Figure 1A.

Figure 1C illustrates typical unrectified EMG recordings (from the same participant as in Fig. 1A) of lower limb muscles while making either brief voluntary ballistic muscle contractions or rapid alternating rhythmic movements. Note that EMG burst duration in rhythmic movements was consistently reduced (vertical dashed lines in Fig. 1C indicate onset and offset of EMG bursts). These data are presented in more detail in Table 1, where the mean EMG burst durations for rapid ballistic and rhythmic movements are listed for each muscle averaged across participants. Although on visual inspection most of our data appeared normally distributed, only ballistic EMG burst durations recorded from cranial muscles and rhythmic EMG burst durations recorded from trunk muscles satisfied the Shapiro‐Wilk test. Nonparametric statistical tests were therefore applied to the data. Analysis of regional data showed that despite rhythmic EMG bursts appearing shorter than ballistic EMG bursts (see Fig. 1C), it was only in the lower limbs that rhythmic EMG bursts were significantly shorter than ballistic EMG bursts (*P* < 0.0001, Wilcoxon signed‐rank test); there was no difference between ballistic and rhythmic trunk or upper limb muscle movements (*P* = 0.88 and *P* = 0.51, respectively, Wilcoxon signed‐rank tests). For ballistic movements, EMG bursts in cranial muscles were significantly longer than those recorded from trunk muscles (*P* = 0.04, Wilcoxon signed‐rank test) and distal arm muscles (*P* < 0.001, Wilcoxon signed‐rank test), but not compared with lower limb muscles.

**TABLE 1:** *Average (and median) ballistic EMG burst durations with standard deviations and number of participants with average EMG bursts of <50 milliseconds*

		Ballistic EMG Bursts						Rhythmic EMG Bursts					
Region	Muscle	Mean (ms)	Median (ms)	Range (ms)	SD (ms)	n < 70 ms	n < 50 ms	Mean (ms)	Median (ms)	Range (ms)	SD (ms)	n < 70 ms	n < 50 ms
Cranial	Temp	141	136	47.9–235.7	40	1/18	1/18						
	OO	122	121	96.1–149.6	19	0/19	0/19						
	Ris	125	115	73.2–187.8	36	0/15	0/15						
Trunk	Trap	101	97	58.6–182	35	5/18	0/18						
	IS	122	114	64.8–245.9	44	1/18	0/18						
	RB	128	108	75.8–264.6	62	0/17	0/17						
	LD	92	94	52.2–151.4	29	4/14	0/14						
	PM	93	95	27.6–138	32	3/15	2/15	105	92	40.2–222.5	47	4/18	1/18
	RAS	189	189	65.1–368.5	80	2/17	0/17	135	137	52.8–209‐3	42	1/18	0/18
	RAI	167	174	63.2–281.2	72	1/16	0/16	115	124	62.5–154.6	26	1/14	0/14
UL	Delt	185	173	49.8–401.9	89	1/35	1/35	62	57	43.2–115.6	22	7/11	4/11
	TB	189	184	67.4–279.2	65	1/18	0/18	140	132	76.4–212.5	40	0/16	0/16
	BB	131	128	68.6–223.1	42	2/20	0/20	190	191	89.5–259.8	54	0/10	0/10
	EDC	102	102	41.5–175	41	6/19	3/19	127	120	45.3–226.8	57	3/15	1/15
	FCU	74	63	36–116.6	29	10/18	4/18	66	64	33.3–112.2	27	6/8	2/8
	APB	106	93	59.4–186	41	4/18	0/18						
	FDI	75	77	40–110.3	19	6/16	3/16						
LL	VL	132	115	40.5–304.3	67	12/63	4/63	91	88	36–235.8	42	16/32	6/32
	BF	178	173	79.8–289.2	57	0/30	0/30	67	70	32–115.9	38	14/27	6/27
	TA	178	168	44.6–360.7	91	4/30	1/30	62	56	23.3–106‐8	20	20/27	8/27
	MG	122	104	23.3–264	75	10/32	7/32	65	64	19.9–127.3	27	17/33	12/33
	EDB	112	116	64–165.8	28	2/18	0/18						
	AH	78	76	40–136	26	7/16	2/16						

Abbreviations: EMG, electromyogram; ms, milliseconds; SD, standard deviation; Temp, temporalis; OO, orbicularis oculi; Ris, risorius; Trap, trapezius; IS, infraspinatus; RB, rhomboid; LD, latissimus dorsi; PM, pectoralis major; RAS, rectus abdominis superior; RAI, rectus abdominis inferior; Delt, deltoid; TB, triceps brachii; BB, biceps brachii; EDC, extensor digitorum communis; FCU, flexor carpi ulnaris; APB, abductor pollicis brevis; FDI, first dorsal interosseous; UL, upper limb; LL, lower limb; VL, vastus lateralis; BF, biceps femoris; TA, tibialis anterior; MG, medial gastrocnemius; EDB, extensor digitorum brevis; AH, abductor hallucis.

Further analysis of appendicular muscles showed that ballistic EMG bursts recorded from proximal muscles compared with distal muscles have a significantly longer duration in both upper limbs and lower limbs (*P* < 0.0001 and *P* = 0.037, respectively, Wilcoxon signed‐rank tests). For rhythmic muscle contractions, EMG bursts recorded from upper limb muscles were significantly longer than those recorded from lower limb muscles (*P* < 0.0001, Wilcoxon signed‐rank test). Although there was no significant difference between the durations of rhythmic EMG bursts recorded from proximal and distal upper limb muscles (*P* = 0.142, Wilcoxon signed‐rank test), bursts recorded from distal lower limb muscles were significantly shorter than those recorded from proximal lower limb muscles (*P* = 0.003, Wilcoxon signed‐rank test).

There was no significant correlation between age and EMG burst duration for ballistic movements, rhythmic movements, or all movements combined (*P* = 0.93, *P* = 0.44, and *P* = 0.40, respectively, Spearman's rank correlation coefficient).

Minimum EMG burst durations for all muscles tested are summarized in Table 1. We observed short‐duration physiological EMG burst durations in a large proportion of our healthy control population. The highest percentage of short EMG bursts was seen in the rhythmic EMG lower limb muscle group with 57% of participants averaging <70 milliseconds. Somewhat unexpectedly, we also observed that 18% of healthy participants could generate average voluntary EMG bursts of <50 milliseconds in duration when contracting muscles rhythmically (see Fig. 1D). As can be seen from Table 1, short‐duration EMG bursts were most frequently observed in the forearm, hand, and lower limb muscles, and occasionally in shoulder muscles (pectoralis major and deltoid). EMG bursts <50 milliseconds were never observed in the orbicularis oculi, risorius, trapezius, infraspinatus, rhomboid, latissimus dorsi, rectus abdominis superior, triceps brachii, biceps brachii, abductor pollicis brevis, or extensor digitorum brevis.

**FIG 2:**
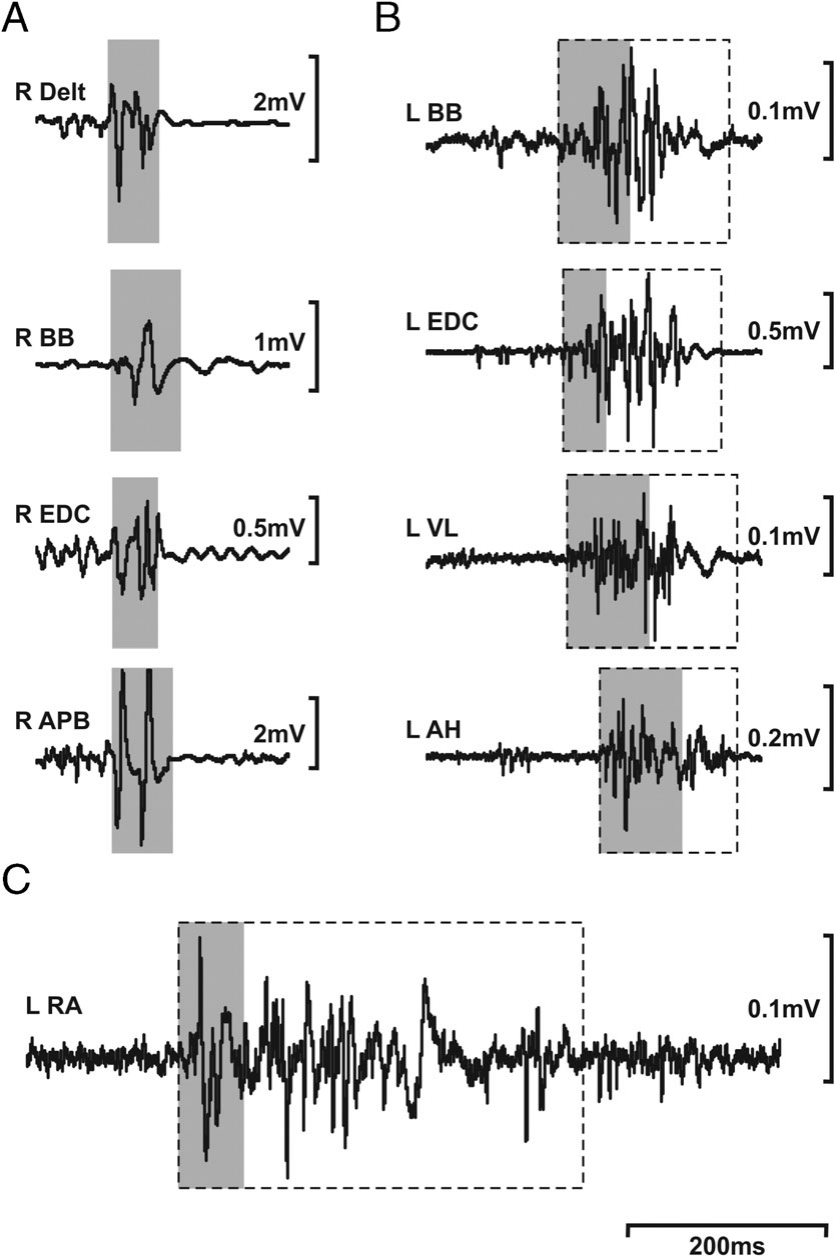
Examples of unrectified surface electromyogram (EMG) recorded from 3 patients with a diagnosis of myoclonus. (**A**) A 48‐year‐old woman with a 5‐year history of action myoclonus after recovering from hypoxic–ischemic encephalopathy. (**B,C**) Two patients with a diagnosis of functional movement disorder: (**B**) a 60‐year‐old man with a psychiatric history of more than 40 years (multiple prolonged admissions with psychosis) and a 2‐year history of relapsing–remitting generalized myoclonic jerks and (**C**) a 64‐year‐old man with a background of chronic right leg pain who developed abdominal myoclonus following 2 significant and simultaneous life events (the death of his mother and the arrest and imprisonment of his son). Dashed boxes indicate the limits of each EMG burst. Gray boxes demarcate the minimum EMG burst duration for each muscle (see Table 1). In (**A**), note that there are no dashed boxes because each EMG burst falls within the gray box. AH, abductor hallucis; APB, abductor pollicis brevis; BB, biceps brachii; Delt, deltoid; EDC, extensor digitorum communis; L, left; RA, rectus abdominis; R, right; VL, vastus lateralis.

As an initial test of the diagnostic utility of the minimum EMG burst duration data collected during this study and presented in Table 1, we reviewed EMG data from a sample of patients with a diagnosis of myoclonus. These results are shown in Figure 2. In a 48‐year‐old woman with a 5‐year history of action myoclonus after recovering from hypoxic–ischemic encephalopathy (the result of an out‐of‐hospital respiratory arrest requiring intubation and prolonged admission to the intensive care unit, ie, Lance‐Adams syndrome; Fig. 2A), the duration of EMG bursts from each muscle was less than the minimum voluntary EMG burst duration for the same muscles recorded from healthy controls (see Table 1). In contrast, in the 2 patients with a diagnosis of FMD, a 60‐year‐old man with a history of recurrent psychotic episodes since his teenage years (each necessitating prolonged admission to a psychiatric facility) and a 2 year history of relapsing–remitting generalized myoclonic jerks (Fig. 2B) and a 64‐year‐old man (Fig. 2C) with a background of chronic right leg pain who developed abdominal myoclonus following 2 significant and simultaneous life events (the death of his mother and the arrest and imprisonment of his son), EMG bursts in the affected muscles were clearly longer than minimum EMG burst durations, as summarized in Table 1.

## Discussion

The primary motivation for this study was to define the physiological limits of EMG burst durations during rapid voluntary movements for a range of muscles, thus providing a comprehensive reference for the neurologist or neurophysiologist investigating complex hyperkinetic movement disorders, particularly FMDs. Our preliminary analysis did not appear to show a significant change in EMG burst duration with age. However, we acknowledge that older age groups were underrepresented in our sample. Future studies of the physiology of EMG burst duration should include a more systematic investigation of the effects of age.

Although we have focused on the utility of minimum EMG burst duration in the clinic, the variability of EMG burst duration might also be a useful clinical measure. Voluntary EMG bursts are generated by engaging a number of different neural pathways, from movement to movement, and are thus highly variable (see Table 1). By contrast, in pathological involuntary ballistic movements, such as myoclonus, which are more stereotyped and generated by a much more limited repertoire of neural pathways, the associated EMG activity should be less variable. However, to our knowledge, this hypothesis has not been investigated formally. The standard deviation of pathological EMG burst duration might therefore be another measurable electrodiagnostic parameter in the investigation of movement disorders and merits further investigation.

Although the prevalence of FMDs is unknown, they are thought to be relatively common; it has been estimated that up to 40% of patients seen in movement disorder clinics are diagnosed with FMD.^7^, ^8^ Although the etiology is not entirely understood, recent accounts have suggested that FMDs arise because of abnormal predictive coding by the brain, specifically miscalibration of internal predictions of the sensory consequences of movement.^10^ Within this conceptual framework, motor manifestations of FMDs remain constrained by physiological limits imposed by motor control circuitry.

Making the correct diagnosis in certain hyperkinetic movement disorders can be challenging, particularly when FMD is within the differential (eg, dystonia,^16^ myoclonus^17^). When FMDs are not recognized, unnecessary and costly investigation ensues,^18^ appropriate early therapeutic interventions may not be provided^19^, ^20^ and of more concern, inappropriate (and occasionally high risk) therapeutic interventions are considered.^21^

EMG burst duration is a simple, widely used guide to diagnosis (and localization) of myoclonus^4^ and has the advantage of requiring little expertise to measure. However, our data suggest that EMG burst duration <50 milliseconds is not a reliable criterion for deciding whether a movement is pathological, particularly when applied to certain muscle groups (as is evident from a previous study^22^).

Our results also provide potential insights into the physiology of motor control. For ballistic contractions in the limbs (see Table 1), minimum EMG burst durations were longer in what are traditionally considered pyramidal muscles (extensor digitorum brevis, tibialis anterior, and extensor digitorum communis) compared with their antagonists (abductor hallucis, medial gastrocnemius, and FCU, respectively). Although voluntary control of the latter group of muscles is mediated to a greater extent by polysynaptic descending pathways that exert both excitatory and inhibitory effects on motoneurons (eg, cortico‐reticulospinal inputs^23^), that of the former is mediated mainly by direct monosynaptic corticospinal connections.^24^ This would suggest that rapid and brief movements are better generated by these polysynaptic descending pathways. Intriguingly, motoneurons controlling PM, which has an exceptionally short minimum EMG burst duration for a trunk/proximal arm muscle (27.6 milliseconds; see Table 1), receive particularly strong cortico‐reticular input.^25^, ^26^

Finally, as noted by others,^1^, ^4^ in the context of FMD it is important that EMG burst duration is not interpreted in isolation and where possible the presence or absence of co‐contraction of agonist–antagonist muscles should also be assessed.

## Author Roles

(1) Research Project: A. Conception, B. Organization, C. Execution; (2) Statistical Analysis: A. Design, B. Execution, C. Review and Critique; (3) Manuscript Preparation: A. Writing of the First Draft, B. Review and Critique.

A.F.C.: 1C, 2B, 3A

S.T.R.B.: 1C

M.R.B.: 1A, 1B, 2A, 2C, 3B

## Disclosures

### Ethical Compliance Statement

The Ethics Committee at Newcastle University approved this study and we conformed to the Declaration of Helsinki. Written informed consent was obtained from all participants in this study on the day of the data collection. We confirm that we have read the Journal's position on issues involved in ethical publication and affirm that this work is consistent with those guidelines.

### Funding Sources and Conflicts of Interest

None of the authors has any conflict of interest to disclose and there was no specific research funding for this project.

### Financial Disclosures for the Previous 12 Months

A.F.C. is employed by the University of Sheffield and has an honorary contract with Sheffield Teaching Hospitals National Health Service (NHS) Foundation Trust. S.T.R.B. was funded by an undergraduate scholarship from INSPIRE (Academy of Medical Sciences/Wellcome Trust) and is currently employed by South Tees Hospitals NHS Foundation Trust. M.R.B. is employed by Newcastle upon Tyne Hospitals NHS Foundation Trust and Newcastle University. His research has been funded by grants from the following organizations: Medical Research Council, the William Leech Charity, National Institute for Health Research, Ataxia UK, Friedreich's Ataxia Research Alliance.
